# Involving the employer to enhance return to work among patients with stress-related mental disorders – study protocol of a cluster randomized controlled trial in Swedish primary health care

**DOI:** 10.1186/s12889-018-5714-0

**Published:** 2018-07-06

**Authors:** Lisa Björk, Kristina Glise, Anders Pousette, Monica Bertilsson, Kristina Holmgren

**Affiliations:** 1 The Institute of Stress Medicine at the Västra Götaland region, Gothenburg, Sweden; 2 The Department of Work Science and Sociology, University of Gothenburg, Gothenburg, Sweden; 3 The Department of Psychology, University of Gothenburg, Gothenburg, Sweden; 4 The Department of Environmental and Occupational Medicine, Institute of Medicine, Sahlgrenska Academy, University of Gothenburg, Gothenburg, Sweden; 5 The Section for Epidemiology and Social Medicine, Institute of Medicine, Sahlgrenska Academy, University of Gothenburg, Gothenburg, Sweden; 6 The Department of Health and Rehabilitation, Institute of Neuroscience and Physiology, The Sahlgrenska Academy at the University of Gothenburg, Gothenburg, Sweden

**Keywords:** Return to work, Common mental disorders, Stress, Coordination, Intervention

## Abstract

**Background:**

Work-related stress has become a major challenge for social security and health care systems, employers and employees across Europe. In Sweden, sickness absence particularly due to stress-related disorders has increased excessively in recent years, and the issue of how to improve sustainable return to work in affected employees is high up on the political agenda. The literature on interventions for return to work in patients with common mental disorders is still inconclusive. This randomized controlled trial (RCT) aims to contribute with knowledge about how physicians and rehabilitation coordinators in primary health care can involve the employer in the rehabilitation of patients with stress-related disorders. The objective is to evaluate whether the early involvement of the patient’s employer can reduce the time for return to work compared to treatment as usual. A process study will complete the RCT with information about what prerequisites primary health caregivers need to succeed with this endeavor.

**Methods:**

Twenty-two primary care centers were randomized to either intervention or control group. At the intervention centers, physicians and rehabilitation coordinators underwent training, providing them with both knowledge and practical tools to involve the employer in rehabilitation. At the patient level, employed patients with an ICD-10 F43 diagnosis were eligible for participation (*n*=132). Difference in proportion of patients on full- or part-time sick leave at three, six and 12 months after inclusion will be investigated. Register data, logbooks and interviews with coordinators and physicians at both intervention and control centers will be used for process evaluation.

**Discussion:**

Although the issue of how to tackle work-related stress can be recognized all across Europe, Sweden face an urgent need to curb the disproportional increase of stress-related disorders in the sick-leave statistics. Since physicians are limited by time constraints, the rehabilitation coordinator may be a helpful resource to take this contact. The current study will contribute to knowledge about how this collaboration can be organized to facilitate employer involvement and reduce time to return to work among patients suffering from work related stress.

**Trial registration:**

Registered on 1 November 2016, ClinicalTrials.gov, NCT03022760.

## Background

Work-related stress occurs when the demands of the job exceed a worker’s ability to cope with them. It is the second most frequently reported work-related health problem in Europe; about one fourth of European workers say they experience work-related stress for all or most of their working time [1]. The considerable costs of stress-related disorders can be found at all levels: loss of health and income for the sick listed employee; loss of productivity, high levels of absenteeism and turnover for the employer and strained health care services and social security systems for the society [2, 3]. To prevent stress at work and to increase work capacity in employees with stress-related disorders is a challenge on a national as well as on the European level.

In Sweden, psychiatric diagnoses are the most common cause to sickness absence (SA), among both women and men [4], and the number of new cases of sick-leave periods^1^ in psychiatric diagnoses rose from 16 cases per 1000 employees in 2010, to 28 cases per 1000 employees in 2016 [5]. Tackling these increasing sick-leave rates is high up on the Swedish political agenda. Exhaustion disorder (ED) is a recognized diagnosis in Sweden since 2005, which justifies compensation from the social security system. Adjustment disorders and reactions to severe stress (Table 1) represent about 50 % of sick-leave cases^2^ due to psychiatric disorders [6].

**Table 1:** The International Classification of Disease 2010 F43: Reaction to sever stress, and adjustment disorders

F43.0	Acute stress reaction
F43.1	Post-traumatic stress disorder
F43.2	Adjustment disorders
F43.8	Other reactions to severe stress
F43.8A^a^	Exhaustion disorder
F43.9	Reaction to severe stress, unspecified

^a^This diagnosis has been added to the Swedish ICD-10 scheme

Work-related factors such as high job demands, low job control, and low social support, increase the risk for sickness absence (SA) due to common mental disorders (CMD) in general [7], and for the development of burnout symptoms in particular [8]. For most people, a period of sickness absenteeism ends with the patient returning to work. Apart from a number of important variables at the individual level, such as symptom severity and a strong expectation to return to work (RTW), workplace level factors such as a sufficient social support from colleagues and supervisor are important determinants for a successful RTW process in CMD patients [7, 9]. Employees with low work demands and a strong RTW self-efficacy and work ability have also been shown to be more likely to RTW after a period of SA due to a CMD [10].

In the past years, several reviews and meta-analyses have been concluding the evidence from a growing number of studies on both clinical and workplace interventions for RTW in patients with CMD. These studies include a plethora of operationalizations of sickness absenteeism and RTW and are conducted on patients with different mental afflictions- most commonly anxiety and/or depression. To reduce sickness absenteeism and/or enhance RTW for workers with specific diagnoses, we find support for problem solving therapy (PST) for people with adjustment disorders [11]; for cognitive behavioral therapy (CBT) and work-directed measures for workers with depression [12]; and physical activity [13] and work-directed interventions (such as exposure therapy, CBT- and PST- based RTW programs) in patients with anxiety and/or depression [14]. In a systematic review and meta-analysis focusing on tertiary interventions in clinical burnout, the authors conclude that successful interventions incorporate the employer in rehabilitation [15].

When CMD is more broadly defined, scholars have recommended a cohesive and work-focused rehabilitation program, involving several stakeholders [16], as well as work-focused CBT and interventions including a combination of health focused interventions, service coordination interventions and work modification interventions [17].

But there are also recent reviews where no, or very limited, support for work-directed interventions for improving RTW in workers with CMD were found [18–20]. Thus, there are no univocal conclusions to be drawn from these reviews and any effort to improve RTW in workers with CMD must be done with a careful consideration to the specific institutional context where it is taking place.

In Sweden, the average time (median for all diagnoses) to end a sick-leave case is 44 days. However, for patients with adjustments disorders and reaction to severe stress it takes on average 57 days to end a sick-leave case [6]. The effect of employer involvement in stress rehabilitation has been studied in a Swedish context, and beneficial effects on RTW have been shown [21, 22], also over time for younger patients [23]. In a recent study, CMD patients (out of which over 70 % suffered from exhaustion or adjustment disorder) receiving primary health care were randomized to either CBT; an workplace intervention including early contact with the employer and graded exposure to the workplace; or a combination of the two [24]. One year after intervention however, there was no difference in days on sick leave between the three treatment groups. Research on workplace involvement in the rehabilitation of patients with stress-related disorders are thus inconclusive also in a Swedish context.

All registered physicians have the right to issue sickness certificates in Sweden, and the vast majority of sick-leave notes are dispensed by general practitioners (GP) within the primary health care system. To provide sufficient and accurate information in the sickness certificate is a challenging task for physicians [25]. In recent years, the economic incentives for the primary health care to involve the employer in rehabilitation have been reinforced. A new function- the rehabilitation coordinator (RC) - has been introduced in all Swedish county councils to support sick-listed patients during their rehabilitation. The very objective with this function is to facilitate patients’ RTW. The RC should also provide the health care system with knowledge about the social insurance system, and coordinate actions between different stakeholders around the patient [26]. The role of the RC is under development and there is yet little guidance on best practice for this new group of primary care professionals [27]. This opens up a window of opportunity to evaluate methods that can be used by the RC to enhance RTW among this large patient group.

Several agreements have been settled at the national level between the government and the Swedish Association of Local Authorities and Regions (SALAR) in order to enhance best practice in primary health care when rehabilitating CMD patients back to work. However, these attempts have been criticized for being ineffective in combatting increasing sick-leave rates [28].

With this study we intend to contribute to knowledge about how primary health care – through the RC- can involve the employer in the rehabilitation of patients with stress-related disorders.

## Methods/Design

### Aim

The overall aim of this study is to evaluate whether a systematic procedure of cooperation between GPs and RCs which involves the employer can reduce the time for RTW in patients with stress-related disorders during a 12-month follow-up period. An intervention group will be compared to treatment as usual (TAU). The primary hypothesis is that an early contact between caregiver, patient and employer can stimulate the employer to take measures at the workplace that advance patients’ RTW. However, we expect this relationship to depend on various circumstances, both at the patient and the organizational level, such as the patient’s symptom severity and motivation to RTW, and the participant GPs’ and RCs’ possibilities to adhere to the intervention.

The second aim of the study is to investigate the organizational prerequisites for primary health care centers (PHCC) to involve the employer at an early stage of rehabilitation. This will be done by a process evaluation, using interviews and register data at the center level.

### Setting

In Sweden, county councils are responsible for providing primary health care. Caregivers that comply with regional standards have the right to provide primary health care, and for a modest fee of € 10, citizens have the right to seek care among both public and private suppliers, funded by public means. One important task for Swedish GPs is to issue the sickness certificates that serve as a basis for social insurance benefit assessments, required from the 8^th^ day of a sick-leave period. A sick-leave certificate must contain a large amount of detailed information, for example about the patient’s diagnosis; functional impairments and activity limitation (work incapacity). The physician can seek advice on appropriate sick-leave durations for different diagnoses in a national decision support, issued by the National Board of Health and Welfare to enhance equal treatment over regions and caregivers. The work capacity must be reduced by at least 25 % for the employee to qualify to sickness benefits. The first fourteen days of a sick-leave period is covered by the employer (except for a first qualifying day), thereafter the Social Insurance Agency takes over the financial responsibility [29]. After 90 days of sick-leave, the agency decides on the right to further payments by assessing the employee’s work capacity in relation to all other work tasks offered by the employer. Further benefits are only granted if there are no such possibilities. The employee has the right to be off duty to try another job, with another employer. At day 180, the employee’s work capacity is evaluated in relation to the entire labour market, but only if it can be assumed that the patient will not be able to return to his or her ordinary job before day 365, or if there are other reasonable conditions that inhibit evaluation. Sickness benefits can compensate for an income loss corresponding to 25 %, 50 %, 75 % or 100 % of the employee´s working hours.

The study is conducted in the Västra Götaland region of Sweden, a large county council covering almost 20 % of the Swedish population with 200 public and private PHCCs. There are about 800 GPs employed at these PHCCs and almost all centers can offer the services of a RC. However, coordinators depend on earmarked national subsidies, and it is common for the coordinator to either work part time as for example a nurse or psychologist, or to work as a coordinator at two or even three centers.

The study is conducted at the Institute of Stress Medicine, Västra Götaland region, and the Department of Work Science and Sociology, University of Gothenburg. It is part of the New Ways research program at the Section for Epidemiology and Social Medicine, Institute of Medicine, Sahlgrenska Academy, University of Gothenburg.

### The RCT

#### Target group, recruitment and randomization

The target group for the intervention was GPs and RCs at the included PHCCs, and by extension their patients, sick-listed from work due to a stress-related diagnosis. The recruitment of PHCCs took place between January and October 2016. A first request to the PHCCs to participate were communicated by telephone calls, e-mails and personal meetings with PHCC managers throughout the Västra Götaland region. In order to attain diversity in terms of geographical location, public or private ownership, center size, and the socioeconomic conditions of the area, interested centers were checked against regional register data. We also considered the number of patients with a F43 diagnosis at the center during one year (2015), the center’s involvement in other research projects, and the extent of RC resources at the center.

Out of the 30 public and private PHCCs that were invited to participate, 22 accepted; 15 public and seven private centers. Managers accepted the center’s commitment by signing a contract with the researchers. Managers were asked to make sure that the entire staff agreed to the center’s participation in the study before signing the contract. The researchers visited each center to inform the staff about the study prior to commitment. PHCCs were offered economic compensation for recruited patients; € 50 per patient in the control group and € 100 per patient in the intervention group. Also, all centers were offered the one day training program, however they were informed that if allocated to the control group, they would have to wait until all patients were recruited to the study.

Based on similar characteristics in register data, we matched centers in pairs. From each pair, one center was randomly selected to the intervention group and the other one to the control group. The staff of the participant centers were all aware about their allocation in the RCT, blinding was not possible in this study.

Employed patients with a F43 diagnosis as the main diagnosis, getting their sickness certification renewed were eligible for participation. Excluded were patients with post-traumatic stress disorder, F43.1. Patients who did not read or speak Swedish, and patients who have had a sick-leave period of more than 60 subsequent days during the past three years before inclusion were not asked to participate.

#### The intervention

The intervention consisted of three components: a one-day training for all participant GPs and RCs, a standardized work procedure for GPs and RCs to follow after training, and the possibility to seek clinical advice from specialist physicians in the research group. The design of the intervention was guided by occupational therapy theory, where occupational performance is regarded as the outcome of a continuous interaction between the person (P), the environment (E) and the occupation (work tasks) (O) [30]. The PEO-model suggests that in order to improve occupational performance, therapists must direct their interventions not only to the individual, but also to the individual’s environment and occupation. In rehabilitation, therapists can assist in for example the adjustment of work tasks, regulation of spare time activities, and modification of the work environment so that the employee can keep work capacity despite physical or mental symptoms and impairments. This requires a shift away from a biomedical perspective to a more holistic approach which takes the patient’s situation into consideration.

In order to enhance knowledge about the relationship between work; stress-related disorders; work capacity and RTW among GPs and RCs, a one day’s training was compulsory for the participants in the intervention group. Lectures and workshops were conducted by researchers who are also experienced clinicians (physicians, psychologists and occupational therapists). The participants were taught both *why* and *how* the employer can be involved at an early stage in the rehabilitation of patients with stress-related disorders. The training day was offered at six occasions from November 2016 to September 2017. GPs (n=66) and RCs (n=13) from the eleven intervention centers underwent training.

The intervention’s central component was a standardized procedure of four steps involving the patient, the GP, the RC and the patient’s employer. This procedure was based on the clinical experience from twelve years of treating patients with exhaustion disorder at the Institute of Stress Medicine [31], and adapted to the primary health care setting, in dialogue with GPs and RCs prior to the intervention. Ordinary medical and psychological treatment and rehabilitation (TAU) was conducted parallel to this procedure.

When a GP had identified a patient for inclusion (step one), the RC set up a meeting with the patient. During this meeting, the RCs informed the patient about the study and collected his or her written consent. The patient filled out a questionnaire containing questions on background characteristics, occupation, symptoms, work stressors and private life stressors, work ability, RTW self-efficacy, employer activities, RTW motivation, and general health (see Table 2). The questionnaire took about 30 minutes to fill in. Patients declining participation were asked to fill in their year of birth, gender and occupation. The RC then used the questionnaire to interview the patient for about one hour (step two), scanned the questionnaire to the medical journal, and provided the GP with a summary. In the next step (step three), the RC called the patient´s employer and filled out a form containing questions about the employer’s view on the situation before and after the patient’s sick-leave, and about the employer’s readiness for the patient’s RTW. This form was also scanned to the medical journal, and the GP was provided with a summary. The procedure ended with a meeting between the RC, the GP, the patient and the employer to set up a plan for RTW (step four). After this final step, the RC filled out a checklist about protocol adherence. All forms and questionnaires also served as research data, and were collected by the researchers.

**Table 2:** Content of the patient questionnaire

Item	Label	Questions & references
Q 1-8	Background characteristics	-
Q 9-12	Exhaustion disorder	Questions from Glise et al. [32] on self-reported exhaustion (s-UMS)
Q 13-50	Work stressors	Items from the Work Stress Questionnaire (WSQ) [33]; Job Control Questionnaire (JCQ) [34]; the General Nordic Questionnaire for Psychological and Social Factors at Work (QPS Nordic 34+) [35]; and the Copenhagen Psychosocial Questionnaire (COPSOQ II) [36]
Q 51-53	Work ability	Items from the Work Ability Index (WAI) [37]
Q 54	RTW- self efficacy	Questions from Lagerveld et al. 2010 [38] on return to work self-efficacy (RTW-SE)
Q 55-57	Employer activities before sick-leave	Questions from the method Workplace Dialogue for Return to Work [21, 39]
Q 58-60	RTW motivation	Item on RTW motivation in van Oostrom et al. 2010 [40]
Q 61a-n	Life stressors	Questions from Hasselberg et al. 2014 [41]
Q 62-75	Symptoms of anxiety and depression	Items from the Hospital Anxiety and Depression scale (HAD) [42]
Q 76-93	Symptoms of burnout	Items from the Shirom-Melamed Burnout Questionnaire (SMBQ) [43]
Q 94	General health	One item from the SF-36 scale [44]

During the intervention, all participants in the intervention group were offered clinical advice by phone or e-mail from the specialist physicians at the Institute of Stress Medicine.

The control group started inclusion at the same day as the first training day for the intervention group, in November 2016. After providing their informed consent, patients at the control centers received TAU, which can include for example medical treatment, and psychological therapy. At some of the control centers, TAU also included RC getting in contact with employers, but normally this is done long into the sick-leave period.

Inclusion of patients lasted from November 2016 until the end of January 2018. The recruitment period was extended by six months compared to the original plan. During the intervention, the researchers revisited all centers for at least one occasion to encourage GPs and RCs to recruit patients. News letters from the project were regularly sent to all PHCC managers and RCs at both intervention and control centers. Also, research assistants contacted all RCs once a month by phone for follow-up.

#### Sample size

**Figure 1:**
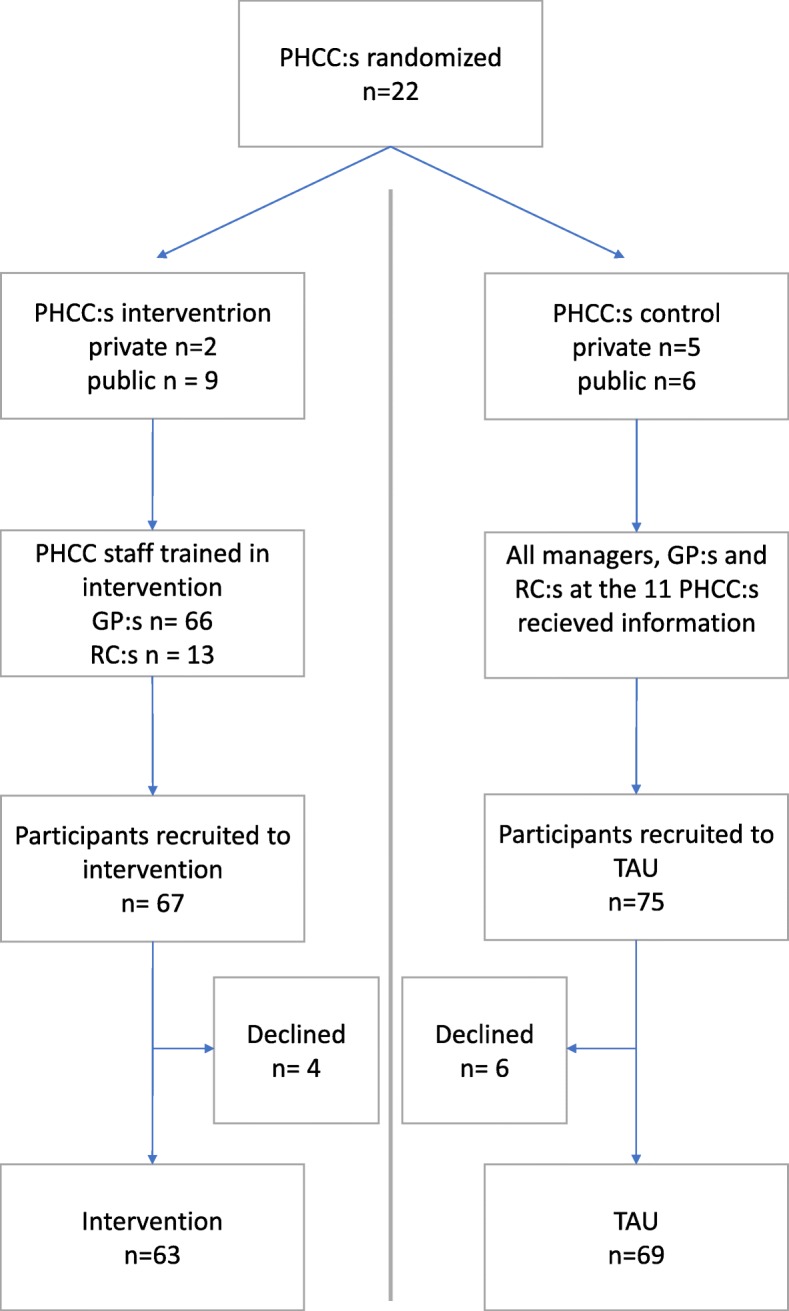
Flow chart of enrolment, allocation and baseline

In all 135 patients were recruited; 69 to the control group and 63 to the intervention group (see Fig. 1). Depending on the proportion of patients on sick-leave at three, six and twelve months after inclusion, power will vary between 41 % and 74 % (based on the assumption that the prevalence of sick-leave in the control group fluctuates between 20 % and 95 % and with an estimated difference between the groups of 15 % [45]; a two-sided test and a statistical significance of p < 0.05). There was a substantial variation in the number of recruited patients per PHCC.

#### Outcome measurements and statistical analyses

The primary outcome of the RCT is RTW. We will analyze the difference between cases and controls in proportion of patients on full- or part-time sick leave at three, six and 12 months after inclusion. We will use data from the Swedish Social Insurance Agency’s Micro Database for Analyzing Social insurance (MiDAS) where all sick-leaves exceeding two weeks are registered. The intention to treat principle will be followed.

The intervention is designed to facilitate for the caregiver to involve the employer in rehabilitation and strengthen the patient’s RTW-SE, but whether these mechanisms are triggered or not will depend on circumstances among patients, employers as well as the caregivers, i.e it will depend on contextual factors [46]. For example, the effect of the intervention on RTW might depend on the patient’s symptom severity, the employer’s readiness for work adaptation, or on the caregivers’ adherence to the protocol. In order to conduct sub-group analysis within the intervention group, a follow-up questionnaire is sent to the patients’ home address by mail at six and 12 months after inclusion. In addition to questions about the rehabilitation process, the questionnaire contains the same items as the baseline measurement. For example we will explore how gender, age, occupation, symptom severity, employer activities and RTW motivation affect RTW.

### The process evaluation

The target of the process evaluation is the participating PHCCs. The register data that was collected in order to select and match centers before randomization were completed with data on work environment, staff composition, staff turnover and quality of care before and during the intervention period. The monthly follow-up phone calls with the RCs were documented in a logbook for each center.

Before intervention, focus group interviews were conducted with GPs, RCs and PHCC managers at the intervention centers (*n*=11). Key questions were about the prevalence of patients with stress-related disorder at the center, and about the tools and procedures when treating and rehabilitating these patients back to work. The GPs’ and RCs’ readiness for change [47, 48] was assessed directly after training.

After intervention, focus group interviews with GPs, RCs and PHCC managers were conducted at 19 out of the 22 participating centers. Participants in the intervention group were asked about how they had organized the implementation, how the intervention had been received among GPs, RCs, and other staff members, and about facilitating and hindering factors for employer involvement in the primary care setting. Participants in the control group were asked similar questions, but with a focus on patient recruitment. In all, 30 interviews were tape recorded and transcribed verbatim.

Together, these data will be used to evaluate the process at the participant centers, and deepen the knowledge about what primary health care needs to involve the employer in the rehabilitation of patients with stress-related disorders.

## Discussion

Given that patients with stress related disorders constitute the largest and fastest increasing group in the sick-leave statistics in Sweden, there is an urgent need to come up with methods to stop this development. Most of these patients get their sickness certificate issued by a GP in the primary health care system. Although current evidence of how to enhance RTW for CMD patients, including stress-related disorders, is yet inconclusive, many recent reviews point to that employer involvement early on in the process might be an efficient way to reduce sick-leave. However, time constraints and uncertainties about the relationships between work and stress hinder many GPs to involve the employer. RCs have been introduced into Swedish primary health care to support the patient in the RTW process through coordination between different stakeholders. This opens a window of opportunity to develop new procedures and tools. This RCT was designed to contribute to knowledge about how procedures of cooperation between GPs and RCs can be organized to facilitate employer involvement and reduce time to RTW among patients suffering from work related stress. Despite considerable efforts to encourage and support GPs and RCs at the participating centers, it turned out to be difficult to recruit patients. Unless the impact of the intervention on RTW is substantial, it will be difficult to detect a difference in RTW between the intervention and control group with the current sample size (*n*= 132).

One reason why earlier studies within this field points to contradictory results [11, 49] might be that RCT studies commonly strive to reduce the variation between study participants and their contextual environment, instead of using these differences to explain how and why the intervention can have different effects on different groups of people. In the current study we instead assumed that implementation would depend on contextual factors at the organizational level, for example on the pre-existing routines, level of corporation and the total workload among GPs and RCs at each center. We also assumed that the impact of the intervention would depend on for example symptom severity and motivation to RTW among patients. The project design was based on these assumptions; numerous centers were recruited, matched and randomized, and patients in the intervention group were asked to reply to several questions at repeated occasions. However, the evaluation of how contextual and individual level factors effect RTW will also be constrained by the small sample size.

Preliminary analyses of the qualitative interviews conducted before and after intervention point to a set of organizational preconditions which need to be in place for GPs and RCs to be able to engage in a successful corporation. The strength of this study will be that the multitude of organizational level data can shed light upon facilitating and hindering factors for employer involvement in the primary care setting. There are many lessons to be learned about how primary care can face the challenging task of employer involvement: What motivates GPs to corporate with RCs? What do RCs need from their organizations to guide patients with stress-related disorders back to work? How can PHCCs ensure routines for sickness certification that enhance RTW? Such knowledge will be valuable for clinicians and policymakers in their future efforts to organize primary health care for improved RTW rates among patients with stress-related disorders. Results from the study will be communicated to the participating centers and other relevant stakeholders, and disseminated through scientific publication.

## Abbreviations

CBT: Cognitive behavioral therapy
CMO: Common mental disorders
ED: Exhaustion disorder
GP: General practitioner
ICD: International classification of diseases
PHCC: Primary health care center
PST: Problem solving therapy
RC: Rehabilitation coordinator
RCT: Randomized controlled trial
RTW: Return to work
RTW-SE: Return to work self-efficacy
SA: Sickness absence
SALAR: Swedish Association of Local Authorities and Regions
TAU: Treatment as usual

## Footnotes

1 ≥14 days

2 Cases of sick- leave periods longer than 14 days which started 2012-2014
